# A Nymphalid-Infecting Group I Alphabaculovirus Isolated from the Major Passion Fruit Caterpillar Pest *Dione juno juno* (Lepidoptera: Nymphalidae)

**DOI:** 10.3390/v11070602

**Published:** 2019-07-03

**Authors:** Bergmann Morais Ribeiro, Ethiane Rozo dos Santos, Luana Beló Trentin, Leonardo Assis da Silva, Fernando Lucas de Melo, Elliot Watanabe Kitajima, Daniel M. P. Ardisson-Araújo

**Affiliations:** 1 Laboratory of Baculovirus, Cell Biology Department, University of Brasilia, Brasilia, DF 70910-900, Brazil; 2 Laboratory of Insect Virology, Department of Biochemistry and Molecular Biology, Federal University of Santa Maria, Santa Maria, RS 97105-900, Brazil; 3 Escola Superior de Agricultura Luiz de Queiroz, University of São Paulo, Piracicaba, SP 13418900, Brazil

**Keywords:** alphabaculovirus, genome, evolution, *Dione juno*, passionfruit

## Abstract

Baculoviruses are capable of infecting a wide diversity of insect pests. In the 1990s, the Dione juno nucleopolyhedrovirus (DijuNPV) was isolated from larvae of the major passionfruit defoliator pest *Dione juno juno* (Nymphalidae) and described at ultrastructural and pathological levels. In this study, the complete genome sequence of DijuNPV was determined and analyzed. The circular genome presents 122,075 bp with a G + C content of 50.9%. DijuNPV is the first alphabaculovirus completely sequenced that was isolated from a nymphalid host and may represent a divergent species. It appeared closely related to Orgyia pseudotsugata multiple nucleopolyhedrovirus (OpMNPV) and other *Choristoneura*-isolated group I alphabaculoviruses. We annotated 153 open reading frames (ORFs), including a set of 38 core genes, 26 ORFs identified as present in lepidopteran baculoviruses, 17 ORFs unique in baculovirus, and several auxiliary genes (e.g., *bro*, *cathepsin*, *chitinase, iap-1, iap-2,* and *thymidylate kinase*). The *thymidylate kinase* (*tmk*) gene was present fused to a *dUTPase* (*dut*) gene in other baculovirus genomes. DijuNPV likely lost the *dut* portion together with the *iap-3* homolog. Overall, the genome sequencing of novel alphabaculoviruses enables a wide understanding of baculovirus evolution.

## 1. Introduction

Baculoviruses are capable of infecting a wide diversity of insect hosts, including larvae of lepidopterans, hymenopterans, and dipterans [1]. The virus plays an ecological role in regulating host populations and is widely used as biocontrol agents in agriculture for pest control [2,3]. Baculovirus infection initiates when the insect feeds upon contaminated food with occlusion bodies (OBs). The OB dissolves and releases occlusion derived-virions (ODVs) in the midgut, which infect epithelial columnar cells. Later on, budded viruses (BVs) are produced from the infected cells and, when they bud from the midgut to the hemocoel, spread the infection throughout the insect body, causing a systemic and lethal disease [4,5]. Infection symptoms for the diseased larvae might include cuticle discoloration, movement loss, reduced feeding, and eventually, depending on the virus species, post mortem liquefaction [6,7]. This is important to guarantee virus spread in the environment [4].

To date, hundreds of baculoviruses have been isolated from different insect species, and many of them have demonstrated a great potential and are successfully used as bioinsecticides in pest management programs [8]. However, taking that insects are the most diverse animals in our planet with more than half of all known animal species [9], an unknown number of insect viruses are still to be discovered. For instance, the discovery of new baculoviruses is important for the understanding of their evolution, host interaction, and consequently the development of new biological control approaches [10,11]. The genome sequence of a virus is one important step for its molecular characterization. In fact, only a few out of those hundreds of known baculoviruses have had their whole genomes sequenced and described. Baculovirus genomes consist of a circular double-stranded supercoiled DNA molecule ranging from 80 to 180 kb encoding 80 to 190 genes [5]. From those, only 38 form a core set of genes shared among all genomes. Based on phylogenetic analysis of the 38 core genes, family *Baculoviridae* is classified into four genera: *Alphabaculovirus* (which contains lepidopteran-specific nucleopolyhedroviruses [NPVs]), *Betabaculovirus* (which contains lepidopteran-specific granuloviruses [GVs]), *Gammabaculovirus* (which contains hymenopteran-specific NPVs), and *Deltabaculovirus* (which contains dipteran-specific NPVs) [1,12].

In a previous work describing the ultrastructure and pathogenesis of a baculovirus isolated from larvae of *Dione juno juno* (Lepidoptera: Nymphalidae), Ribeiro et al. [13] found that *Dione juno* nucleopolyhedrovirus (DijuNPV) was able to effectively kill caterpillars from the species *D. juno* and *Agraulis vanilae* (Lepidoptera: Nymphalidae), as confirmed by Rodrigues et al. [14]. *D. juno* is the most recurrent pest of passionfruit crops in Brazil and other tropical countries, and it eventually causes high damage to crop due to its gregarious habits [15]. *D. juno* caterpillars remain near each other during development, and after destroying leaves, the larva may scrape tissues and buds, which causes delays in plant grow and eventual death. Passionfruit (*Passiflora spp.*) cultures presents a high commercial value worldwide. In Brazil alone, the entire production of passionfruit in 2017 was around 550,000 tons with a rising planting area of 41,000 ha [16]. Several natural biocontrol agents comprising spiders and insects aid in the effective control of insect pests and can be eradicated when unspecific pesticides are applied upon the crop [17]. Hence, the use of discriminatory pesticides such as natural pathogens like bacteria, fungi, and viruses are effective to shield the crop and kill only the targeted caterpillars [18]. Nevertheless, few discriminatory bioinsecticides are available for passionfruit crop pest management and the Bacillus thuringiensis is solely advised for extensive areas [19]. In this study, the complete genome sequence of a novel viral bioinsecticide DijuNPV was determined by two complementary sequencing approaches and analyzed. Phylogenetic and other *in silico* analyses suggested that this virus is a novel and diverse virus that could represent a new species inside genus *Alphabaculovirus*, specifically inside the Group I-forming clade. Moreover, we performed genomic and gene content analysis and also ultrastrutural analysis of purified OBs and OBs in the insect fat body by transmission electron microscopy.

## 2. Materials and Methods

### 2.1. Virus Purification and Insects

Insect cadavers of species *D. juno juno* with symptoms of baculovirus infection were collected from passionfruit plantations at Araguari city (State of Minas Gerais, Brazil) in 1983. 20 g of insect cadavers were used for OB purification [20]. *D. juno juno* eggs were collected in backyard passionfruit vines in Brasilia (Brazil). *D. juno juno* eggs are usually laid in large groups (up to 50), and the caterpillars, after hatching, always live in groups. Larvae were reared on leaves of freshly harvested young passion fruit leaves and kept in cardboard boxes. To amplify the virus, the passionfruit leaves were treated with 1 M Na_2_CO_3_, twice in 70% ethanol, rinsed in ddH_2_O, dried at room temperature, and then exposed to 20 min UV light. The leaves were sprayed with DijuNPV OBs and given to hundreds of newly hatched larvae of *D. juno juno* and kept at 28 °C. After the contaminated leaves were consumed, fresh passionfruit leaves (previously treated with 1 M Na_2_CO_3_ and UV light) were given to the larvae until death.

### 2.2. Transmission Electron Microscopy

The fat body was removed by dissection from infected larvae at 6 d after infection and immediately immersed for 2 h in a mixture of 2% glutaraldehyde and 2% paraformaldehyde in 0.05 M cacodylate buffer (pH 7.2) for tissue fixation. They were then post-fixed in 1% OsO_4_ in the same buffer for 1 h, dehydrated in acetone, and embedded in low viscosity Spurr’s epoxy resin. Blocks were sectioned in a LKB ultratome III ultramicrotome equipped with a Diatome diamond knife and the sections, contrasted with 3% urany1 acetate and Reynold’s lead citrate, were examined in a JEOL JEM l00C transmission electron microscope.

### 2.3. Genome Sequencing, Assembly, and Annotation

DijuNPV genomic DNA was sequenced with two different high-throughput sequencing approaches: the 454 Genome Sequencer (GS) FLX™ Standard (Roche) at the ‘Centro de Genômica de Alto Desempenho do Distrito Federal’ (Center of High-Performance Genomic, Brasilia, Brazil) and the Illumina HiSeq™ 2000 platform at Macrogen Inc. (Seoul, Republic of Korea). The 454 sequencing data was assembled *de novo* using Geneious 9.0 [21], and the *in silico*-predicted restriction enzyme digestion profile was compared to Ribeiro et al. [13]. The open reading frames (ORFs) that started with a methionine codon (ATG) and encoded polypeptides of at least 50 amino acids were identified with Geneious 9.0 and annotated using BLASTX [22]. An acceptable overlap of less than 50% of the ORF within the neighbor ORFs was considered using a more liberal ORF annotation criterion based on Ref. [23]. Tandem Repeats Finder (http://tandem.bu.edu/trf/trf.html) [24] implemented in Geneious 9.0 was used to locate repeat regions. The genomic DNA sequence was submitted to the GenBank with the accession number MK558262.

### 2.4. Phylogenetic Analyses

For the baculovirus phylogenetic analysis, the Multiple Alignment using Fast Fourier Transform (MAFFT) [25] was carried out with concatenated amino acid sequences of 38 baculoviral core genes from 97 publicly available baculovirus genomes (Table S1). A maximum likelihood tree was inferred using the Fast-tree method [26] and a Shimodaira-Hasegawa-like test [27]. For the *iap-3* and *tmk* genomic context comparison, we selected the context between both *ac30-like* and *fgf* orthologs and displayed ORF orientation with colored arrowheads and identified orthologs with similar color and autapomorphies acquisitions with black arrowheads. MAFFT alignments of 38 sequences (for the *cp016*-like genes, whose homolog is DijuNPV-ORF-126) were used with the PHYML method [28] and bootstrapped with 100 repetitions implemented in Geneious R9. The evolution model was predicted by MEGA 7 [29] as Jones–Taylor–Thornton (JTT). The tree for the *cp016*-like gene was midpoint-rooted using FigTree v1.4.0. All the alignments are available upon request.

## 3. Results

### 3.1. Ultrastructure of DijuNPV Occlusion Bodies (OBs)

**Figure 1:**
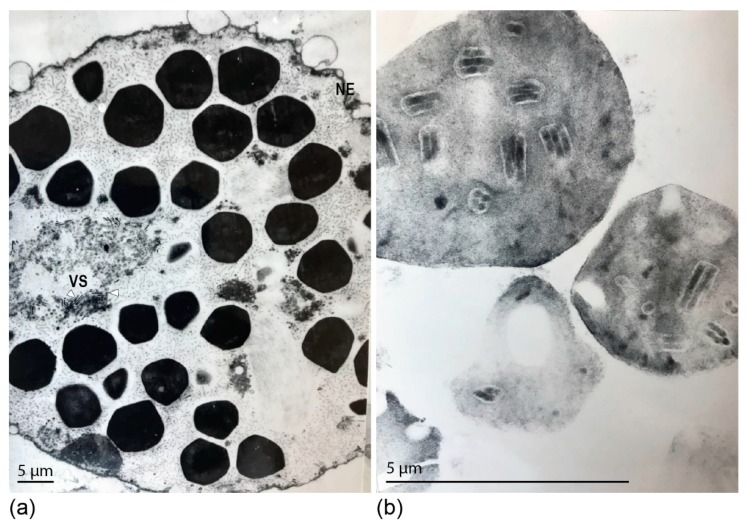
Ultrastructural analysis of the *Dione juno* nucleopolyhedrovirus (DijuNPV) occlusion bodies (OBs). (**a**) Passionfruit caterpillars were fed on contaminated passionfruit leaves, and when the first symptoms of infection appeared, the insects were frozen and dissected for fat body extraction. The tissue was fixed and prepared for transmission electron microscopy. A section of a single cell inset on the nucleus is presented here. The nuclear envelope (NE) surrounds several polyhedral OBs. The virogenic stroma (VS) presents non-enveloped rod-shaped nucleocapsids (white arrowhead). (**b**) Cross section of a purified DijuNPV OB showing several occlusion-derived viruses containing multiple nucleocapsids.

We evaluated the DijuNPV infected fat body cells and the purified OBs by transmission electron microscopy (TEM). The OBs inside infected cells were polyhedral (Figure 1a, black arrowhead). The infected cell nucleus was hypertrophied and presented a discrete virogenic stroma with several non-enveloped nucleocapsids, as characteristic of alphabaculovirus infections (Figure 1a, white arrowhead). Purified OB sections revealed ODVs with multiple nucleocapsids within (Figure 1b). The presence of occlusion bodies in several tissues of the insect body, including midgut and fat body, indicates that the virus is likely polyorganotropic (data not shown).

### 3.2. General Features of the DijuNPV Genome Sequence

We sequenced the DijuNPV genome by two different and complementary high-throughput sequencing approaches: the 454 pyrosequencing and the Illumina HiSeq. Reads from the 454 pyrosequencing were assembled into one single circular genome contig of 122,075 bp with a coverage of 24 ± 9.3 times. The size of the genome and the G + C content (50.9%) were in a range similar to other alphabaculovirus genomes. We annotated 153 ORFs and found two types of repeat regions in the DijuNPV genome, *direct repeats* (*dr1* and *dr2*) and *homologous regions* (*hrs*) (Table S2). We found five *hrs* and divided them into *hr1* (*a* and *b*) and *hr2* (*a*, *b*, and *c*) with sizes ranging from 431 to 1336 bp. The two direct repeat regions consist of the same repeated sequence: *dr1* is located inside the *pp78/83* and presents several small repeat sequences of 10 nt; *dr2* is located inside the *vp80* and presents four repeats of 68 nt and global pairwise identity of 98%. The *hrs* present high pairwise nucleotide identity: *hr1a* and *hr1b* present 88.2%; whereas *hr2a*, *hr2b*, and *hr2c* present 89%. Considering the entire genome, the most abundant short core repeats along the *hrs* were TTACGAGAACATT (31 times), GTACTCGAAAA (30 times), AAAATAGAACA (35 times), and TTTTTAGCGATG (40 times) (Figure S1). Moreover, the location of some DijuNPV *hrs* varied along the genomes of some related viruses, such as OpMNPV and AnpeNPV. However, when compared to the OpMNPV, most of the *hr* loci were maintained. The genomes presented strict collinearity when compared to each other. Interestingly, we also confirmed the restriction enzyme profile of DNA from DijuNPV published by Ribeiro et al. [13] with the *in silico*-predicted restriction enzyme digestion profile (not shown). The approximate average size of the DijuNPV genome predicted based on six restriction profiles was 110 kbps.

### 3.3. Sequence Variation in the DijuNPV Virus Population

We found 203 single nucleotide variants (SNVs) in the Illumina data (about 13 million reads with an average size of 76 nt) mapped against the DijuNPV genome assembled from the 454 sequencing data (data not shown). Most of the variations (180 SNVs) were found inside the coding regions at an average frequency of 38 ± 4.6%. Among them, 88 were found to be non-synonymous (Table S3). The highest-frequency non-synonymous SNVs in the DijuNPV assembly occurred at nucleotide positions 81,296 (45.5%) in the *late expression factor 9* (*lef-9*) gene, 94,838 (47.9%) in the *protein kinase interacting protein* (*pkip*) gene, and 120,022 (45.9%) in the *ac5-like* gene. Ninety-two polymorphisms were synonymous, and no indels were found among the mapped reads. The genes with highest amount of SNVs were *dna-pol* and *gp37* with 10 variations each. For the *dna-pol* coding DNA sequence (CDS), three SNVs were non-synonymous, whereas for *gp37*, six had an impact on the protein sequence (Table S3).

### 3.4. Phylogeny of DijuNPV

**Figure 2:**
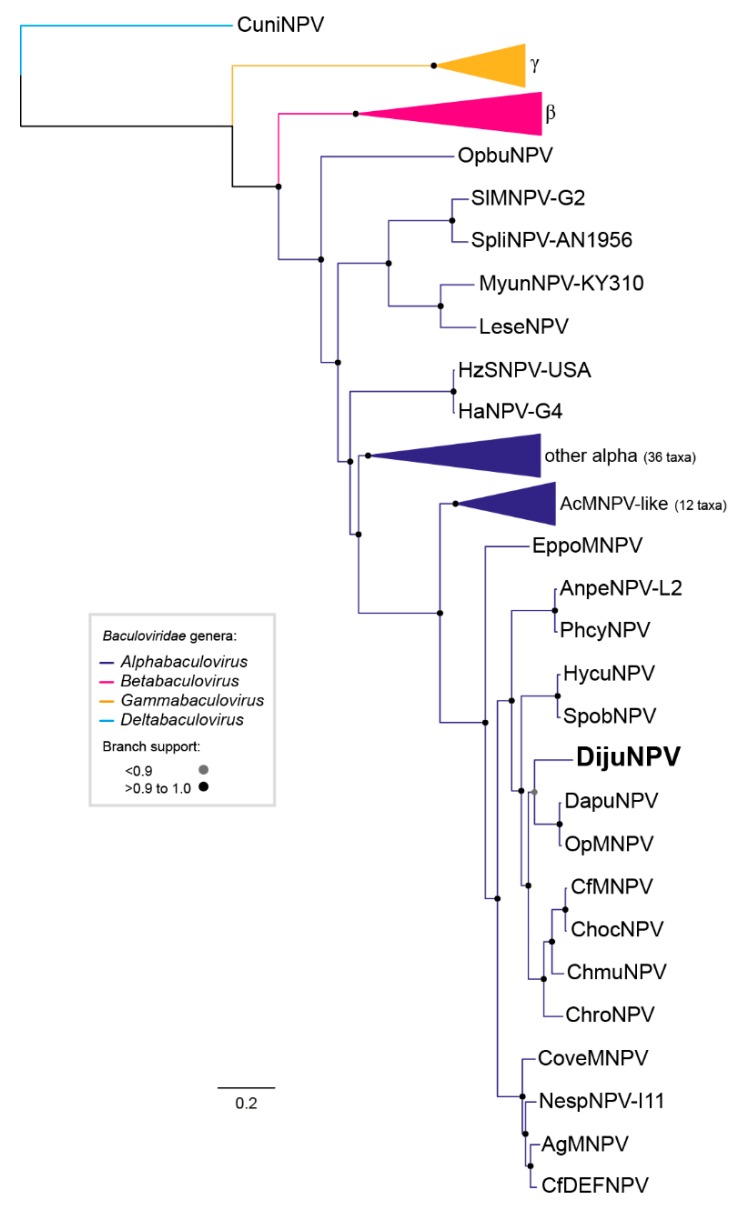
DijuNPV is an alphabaculovirus. Maximum likelihood inference based on the concatenated amino acid sequences of 38 core genes of all complete baculovirus genomes (Table S1). The branch support was determined by the Shimodaira-Hasegawa-like method. Some branches were collapsed for clarity: members inside genus *Gammabaculovirus* (orange), members inside genus *Betabaculovirus* (pink), and members inside genus *Alphabaculovirus* (dark blue). The deltabaculovirus CuniNPV was used as the root (light blue). We collapsed gammabaculovirus, betabaculovirus, some alphabaculovirus, and the Autographa californica multiple nucleopolyhedrovirus (AcMNPV)-like viruses.

We investigated the evolutionary relationship of DijuNPV to other baculoviruses. The baculovirus phylogenetic tree is shown in Figure 2. The current tree topology of baculovirus was observed when DijuNPV sequences were added to the baculovirus dataset. From the tree, DijuNPV appeared as an alphabaculovirus most closely related to OpMNPV, Dasychira pudibunda nucleopolyhedrovirus (DapuNPV), Choristoneura rosaceana nucleopolyhedroviruses (ChroNPV), Choristoneura murinana nucleopolyhedrovirus (ChmuNPV), Choristoneura fumiferana multiple nucleopolyhedrovirus (CfMNPV), and Choristoneura occidentalis nucleopolyhedroviruses (ChocNPV), and a lesser extent to Autographa californica multiple nucleopolyhedrovirus (AcMNPV)-related viruses. The average nucleotide identities based on the concatenated core gene pairwise alignment of DijuNPV to other sequenced alphabaculovirus can be found in Table S1. The phylogeny was inferred based on the 38 conserved genes for several completely sequenced baculoviruses.

### 3.5. DijuNPV May be a Representative of a Novel Species Inside Genus Alphabaculovirus

To check whether DijuNPV may represent a member of a novel species into genus *Alphabaculovirus*, the partial sequences of *lef-8*, *lef-9*, and *polh* pairwise distances derived from the DijuNPV genome and other members that represent other recognized alphabaculovirus species were compared. The aligned sequences are well in excess of 0.05 substitutions/site, fulfilling the criteria to establish a novel species [30]. A wider dataset of those partial genes is available in the GenBank platform associated to both Refs. [30] and [14]. This includes viruses isolated from subjects of the species *Agraulis spp*. and *D. juno*. When a pairwise distance by the Kimura-2-parameters (K2P) model was performed using those partial genes that include viruses isolated from *A. spp.* and *D. juno*, the viruses were found to be isolated from the same virus species as that represented by DijuNPV. Therefore, since the oldest publication [13] for this virus had named the isolate based on the *D. juno* host, we maintained this tentative name of this novel species as *Dione juno nucleopolyhedrovirus*.

### 3.6. Genomic Analysis

**Figure 3:**
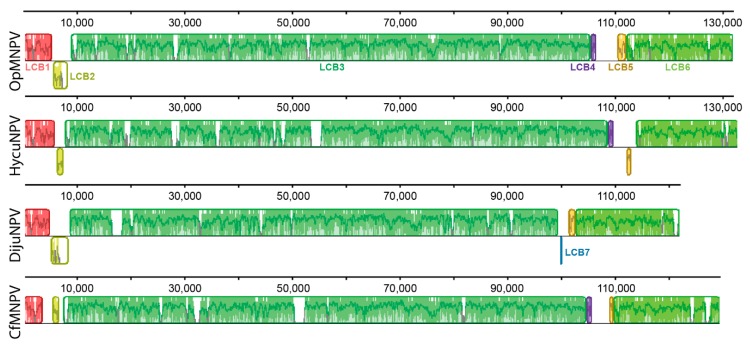
Genome comparison of DijuNPV and its related viruses. DijuNPV is compared to three alphabaculovirus genomes, including Hyphantria cunea nucleopolyhedrovirus (HycuNPV), Orgyia pseudotsugata multiple nucleopolyhedrovirus (OpMNPV), and Choristoneura fumiferana multiple nucleopolyhedrovirus (CfMNPV). The same colors depict the same LCBs across the genomes. Seven Locally Collinear Blocks (LCB) numbered from 1 to 7 were found. The DijuNPV genome lost LCB4 (purple) and present LCB7 (blue) instead. The white regions depict sequence loss.

We performed a genomic comparison among some of the DijuNPV-related virus genomes using a progressive MAUVE algorithm, including Hyphantria cunea nucleopolyhedrovirus (HycuNPV), OpMNPV, and CfMNPV. Seven Locally Collinear Blocks (LCB) were found, and most were shown to be strictly conserved among these genomes (Figure 3). LCB1 and LCB3 are the most conserved blocks among the related viruses. An autapomorphic inversion was found in CfMNPV LCB2. LCB4 was lost by the DijuNPV genome. This region in the other genomes contains the *iap-3* ortholog. When the CfMNPV genome was not included, the DijuNPV genome presented strict collinearity with the OpMNPV and HycuNPV genomes (data not shown).

### 3.7. DijuNPV ORF Content

The DijuNPV genome contains the 38 currently defined core genes [12,31] and the 26 ORFs identified as present in genomes of alpha- and betabaculovirus [31]. Seventeen ORFs presented no homologs in any other baculovirus genomes and did not exhibit no sequence similarity with described domains in HHpred and SMART analyses. Several auxiliary genes were found in the DijuNPV genome, including three copies of the *baculovirus repeated ORF* (*bro*) multigene family called by *bro-a* (DijuNPV-ORF-95), *bro-b* (DijuNPV-ORF-96), and *bro-c* (DijuNPV-ORF-148), *cathepsin* (DijuNPV-ORF-37), and *chitinase* (DijuNPV-ORF-38) that are related to virus horizontal transmission; genes associated with apoptosis control such as *iap-1* (DijuNPV-ORF-120) and *iap-2* (DijuNPV-ORF-88); and genes related to nucleotide metabolism like a *thymidylate kinase* (DijuNPV-ORF-126). Importantly, as characteristic of all group I alphabaculoviruses, the DijuNPV genome presents a *gp64* ortholog (DijuNPV-ORF-36).

**Figure 4:**
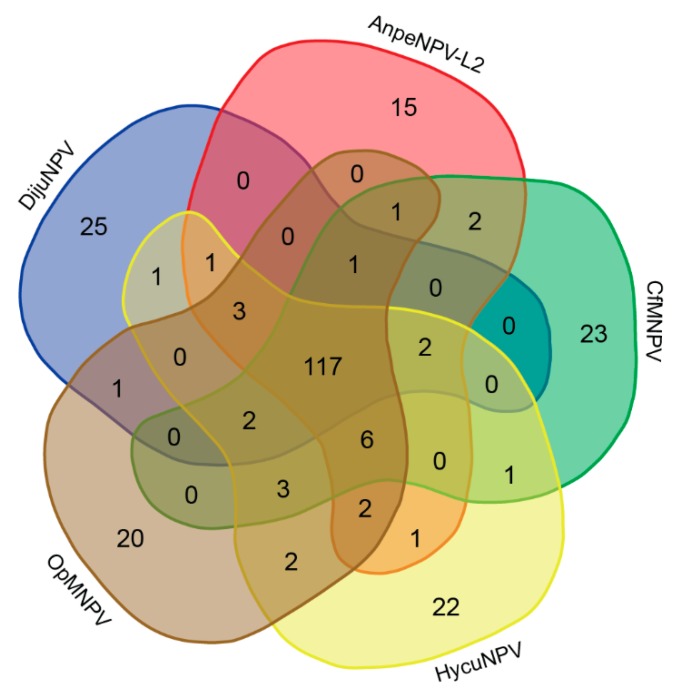
Venn diagram comparing the gene content among DijuNPV and some selected relatives. Total number of genes of the five alphabaculovirus (DijuNPV, AnpeNPV-L2, CfMNPV, OpMNPV, and HycuNPV) were compared by BLASTX to find homologies among the genes of the different genomes. A total of 251 genes were found, 25 were present only in the DijuNPV genome (eight are found in other baculovirus, and 17 are unique to DijuNPV); 117 are shared between the five viruses and only one single gene is found between DijuNPV and OpMNPV, the *tmk* homolog, which in the OpMNPV genome is found fused to the *dut*.

We also performed an ORF content comparison among DijuNPV and its closest relatives (i.e., AnpeNPV-L2, CfMNPV, OpMNPV, HycuNPV), and we plotted the result in a Venn diagram (Figure 4). A total of 251 different genes were found considering all five species. For this comparison, we re-annotated the five genomes under the same criterion and found 36 new genes not annotated before, including 11 in the CfMNPV genome, six in the OpMNPV, five in the AnpeNPV-L2, and 14 in the HycuNPV. Only 117 genes were shared among the five species. Twety-five ORFs were found only in the DijuNPV genome: 17 unique in baculovirus, one baculovirus repeat ORF a (*bro-a*), an *ac45-like* homolog, and other six hypothetical genes found in other baculovirus genomes, including DijuNPV-ORF-4, ORF-9, ORF-10, ORF-25, ORF-75, and ORF-124. The best hits found for each gene is shown in Table S2. We found only one gene shared exclusively by DijuNPV and HycuNPV genomes, which is the *bro-c* (DijuNPV-148). When compared by pairwise alignment, this gene presents a deletion at the amino-terminal portion in the DijuNPV homolog (data not shown). A *tmk* homolog (DijuNPV-ORF-126) is shared only by DijuNPV and OpMNPV, which will be discussed later on. Interestingly, a *conotoxin-like 2* (*clt-2*) homolog was found in the DijuNPV, OpMNPV, HycuNPV, and AnpeNPV-L2 genes and lacked by CfMNPV, whereas a *ctl-1* homolog was found in all related viruses but not in the DijuNPV.

### 3.8. The tmk Homolog Locus

**Figure 5:**
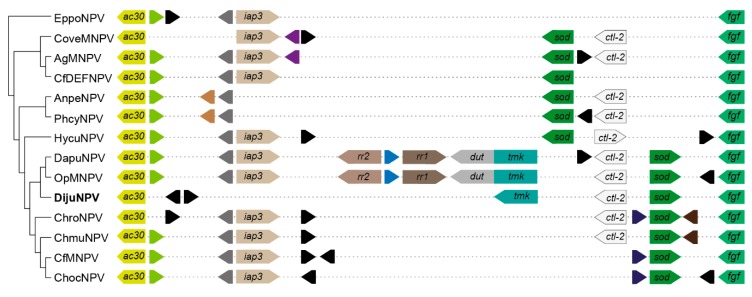
Syntenic genomic context chart containing both *iap3* and *tmk* orthologs in DijuNPV-related species. We show the genomic context together with the phylogeny among the species. The arrowhead shape represents gene orientation, and similar colors represent ortholog genes. Autapomorphies are colored black, and dashed lines concatenate the genes.

**Figure 6:**
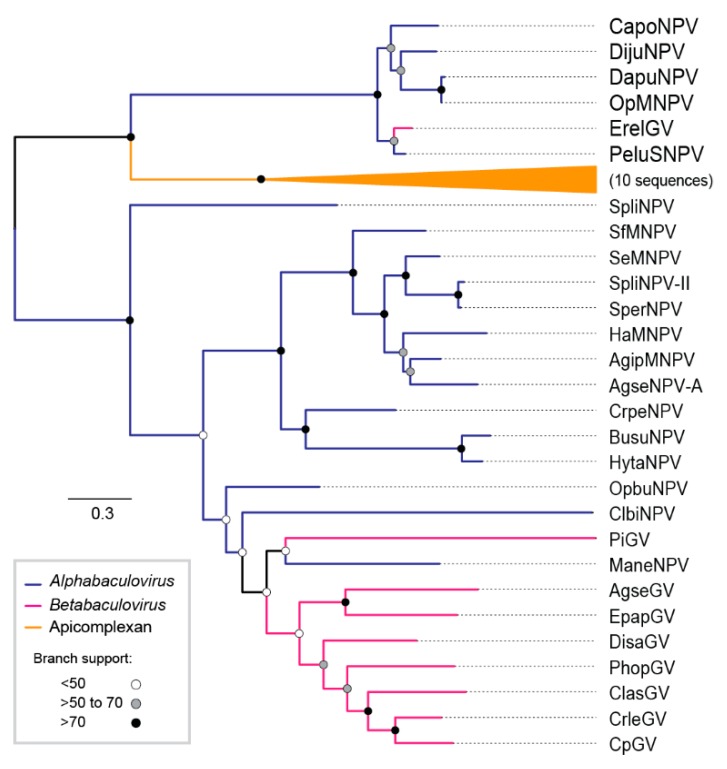
Phylogeny of a putative thymidylate kinase homolog found in the DijuNPV genome. Phylogeny of DijuNPV-ORF-126, a homolog of cp016-like. Erinnyis ello granulovirus (ErelGV), OpMNPV, Dasychira pudibunda nucleopolyhedrovirus (DapuNPV), Perigonia lusca single nucleopolyhedrovirus (PeluSNPV), Catopsilia pomona nucleopolyhedrovirus (CapoNPV), and DijuNPV-derived proteins clustered together, indicating common ancestry. The tree was rooted at the midpoint for clarity.

The loci flanked by *ac30-like* and *fgf* gene orthologs carry the *iap-3* homolog gene in other DijuNPV relatives (Figure 5). However, DijuNPV presents only *iap-1* and *iap-2*, and no *iap-3* homolog was found. DijuNPV seemed to have lost the *iap-3* homolog during evolution (Figure 5) in a similar way to that observed for the AnpeNPV genome. The same locus missing the *iap-3* homolog in the DijuNPV genome, in relation to its relatives, contains a *tmk* homolog (*cp016-like*). The OpMNPV and DapuNPV genomes contain four putative nucleotide metabolism-related genes, including *ribonucleotide reductase 1* (*rr1)*, *ribonucleotide reductase 2* (*rr2)*, and a fusion of *tmk* and *dut*. By alignment and phylogenetic analysis, we found that the DijuNPV *tmk* (ORF-126) was related directly to the *tmk* portion of the fused genes found in both OpMNPV and DapuNPV genomes (Figure 6). In the *tmk* dataset, we included genes related to several baculoviruses and to the mealworm disease-associated apicomplexan *Gregarina niphandrodes*, which were retrieved by BLASTX. The DijuNPV *tmk* clustered together with Erinnyis ello granulovirus (ErelGV), OpMNPV, DapuNPV, and Perigonia lusca single nucleopolyhedrovirus (PeluSNPV), suggesting a common ancestry (Figure 6). The closest relatives were the apicomplexan genes. Betabaculovirus-derived *tmk* genes (except ErelGV) clustered together, and the same occurred with alphabaculoviruses except for Malacosoma neustria nucleopolyhedrovirus (ManeNPV) and Operophtera brumata nucleopolyhedrovirus (OpbuNPV), which clustered together with betabaculoviruses. Therefore, the DijuNPV genome seemed to have lost a region that would harbor homologs of *iap-3*, *rr1*, *rr2*, and *dut*, retaining only the *tmk* homolog.

## 4. Discussion

DijuNPV is highly pathogenic to nymphalid caterpillars of both species *D. juno* and *A. vanilla* and could be potentially used for passionfruit crop protection [13,14]. The isolate of DijuNPV was previously characterized at ultrastructural, biological [13], and molecular [14] levels, though it was never characterized at a wide genomic approach. In this work, we sequenced and characterized the genome of DijuNPV by two deep sequencing complementary techniques (i.e., 454 and Illumina). This is the first baculovirus completely sequenced that was isolated from a nymphalid host. Family Nymphalidae is one out six currently defined butterfly families (i.e., Hesperiidae, Lycaenidae, Nymphalidae, Papilionidae, Pieridae, and Riodinidae) in order Lepidoptera [32]. Other baculoviruses with completely sequenced genomes were isolated from other butterfly families, including Urbanus proteus nucleopolyhedrovirus (UrprNPV) from a hesperiid host [33], Catopsilia pomona nucleopolyhedrovirus [34], three isolates of Pieris rapae granulovirus [35], and Neophasia sp. nucleopolyhedrovirus (unpublished) from pierid hosts.

We found that DijuNPV is an alphabaculovirus specifically related to the still monophyletic group I inside genus *Alphabaculovirus* [33,36]. Group I members are characterized by having a *gp64* homolog as the major BV envelope fusion protein that replaced the ancient *f protein* during clade divergence [37]. The statistical value to support the relationship branch of DijuNPV to its relative viruses is low, which means that its position might be different as other closely related viruses are introduced into the dataset, as observed for another butterfly larvae-isolated baculovirus, i.e., UrprNPV [33]. The branch length separating DijuNPV from the other completely sequenced alphabaculoviruses is in a range that is comparable to the branch lengths separating viruses that are members in other recognized species. Baculoviruses closely related to DijuNPV were isolated from hosts that belong to families Tortricidae, Saturnidae, and Lymantriidae. Those families belong to the same clade called by Ditrysia, whose characteristic is the presence of two distinct sexual openings, one for mating and the other one for laying eggs [38].

We annotated 153 genes in the DijuNPV genome. Baculovirus genes are divided into regulatory, structural, and accessory proteins according to its functional characterization in members of model types, for instance Autographa californica multiple nucleopolyhedrovirus (AcMNPV) [5] inside species *Autographa californica nucleopolyhedrovirus*. Gene functions tend to be extended to homologous proteins in less studied viruses, such as DijuNPV. Despite the diversity in gene content and organization of baculovirus genomes, a set of 38 core genes are conserved across their genomes and play important roles in the viral cycle [12,31]. Surprisingly, DijuNPV likely lost an *iap-3* homolog that is present in other closely related viruses. Although other *iap* genes is found in the alphabaculovirus genomes such as *iap-1* and *iap-2*, usually the product of *iap-3* is the functional IAP that plays role in blocking apoptosis during baculovirus infection [39]. Only AnpeNPV iap-1 was able to block cell apoptosis induced by actinomycin D treatment and also rescued the *p35*-deficient AcMNPV to replicate in Sf9 cells. Based on that observation, it would be valuable to characterize the ability of *iap-1* and *2* products to block apoptosis. Importantly, in our work, we used a more liberal ORF annotation criterion (i.e., accepting an overlap less than 50% of the ORF within the neighbor ORFs) that allowed finding 17 unique genes not reported before in baculovirus. This is criterion is justified based on the empirical data found in Ref. [23]. Many published baculovirus genome analyses do not define the exact minimal overlap between ORFs. A study on the Spodoptera exigua multiple nucleopolyhedrovirus defined the overlap as less than 25 codons (<75 nt). Following this criterion, only three out of 17 would be not annotated as a putative ORF, including ORF43, ORF 70, and ORF146.

Interestingly, the OBs from DijuNPV were isolated in-field and represent a population of viruses. Therefore, the sequencing data generated by the Illumina HiSeq were used to search for intrapopulation virus diversity. We found several synonymous and non-synonymous SNVs in the DijuNPV virus population at an average frequency of 38 ± 4.6 %. In a similar way, the sequencing of an AcMNPV isolate generated identified 118 SNVs with average frequencies of 33–36% [40]. In contrast, variations were present at very low frequencies within the consensus sequence of Operophtera brumata nucleopolyhedrovirus isolate MA (OpbuNPV-MA) when compared to both DijuNPV and AcMNPV, with only ten SNVs occurring at a frequency of ≥8% and most occurring at frequencies of ≤6%.

At the same locus in which the *iap-3* homolog should be found in the DijuNPV, there is a homolog of *tmk*. In a previous work, we described the evolutionary history of this *tmk* homolog (*cp016* homologs). The gene might be found in three different manners in baculovirus genomes: fused to either a *polynucleotide kinase 3*′-*phosphatase* (*pnk*, previously annotated as *nicotinamide riboside kinase* 1, *nrk-1* or *histidinol phosphatase, hisp*), a *dut* homolog, or alone. In alphabaculoviruses, the gene is usually fused to the N-terminal portion of *pnk*, and the unique exception takes place in the *Clanis bilineata* nucleopolyhedrovirus (ClbiNPV) genome where no *pnk* is found. Nucleotide metabolism-related genes are not essential for baculovirus infection given that several species lack them [41]. However, the independent and recurrent acquisition of those enzymes suggests that there is a selective advantage for viruses by means of providing accelerated infection and progeny production [41]. For instance, two recombinant AcMNPV viruses containing homologs of the *tmk-dut* from PeluSNPV and ErelGV were shown to increase viral DNA replication, virus progeny production, and occlusion body formation during in vitro infection when compared to the parental AcMNPV virus that lacks *dut* and *tmk* genes [41].

## 5. Conclusions

In this work, we have described the genome of the first baculovirus isolated from a nymphalid host, the passionfruit pest *D. juno*. The virus may represent a novel species into genus *Alphabaculovirus*, which is closely related to other group I members. The genome was shown to have five *hrs* and 153 ORFs with 17 as unique. Furthermore, several auxiliary genes were encountered, such as homologs of *iap-1* and *2*, *chitinase*, *cathepsin*, *gp37*, and *tmk*. The later was present fused to a *dut* gene in other baculovirus genomes. DijuNPV lost the *dut* portion together with the usually functional *iap-3* homolog. Overall, the genome sequencing of novel alphabaculoviruses enables a wide understanding of baculovirus evolution.

## Supplementary Materials

Supplementary materials can be found at https://www.mdpi.com/1999-4915/11/7/602/s1.
