# Supplementary material for: A Nymphalid-Infecting Group I Alphabaculovirus Isolated from the Major Passion Fruit Caterpillar Pest Dione juno juno (Lepidoptera: Nymphalidae)
Source: Viruses. 2019 Jul 3;11(7):602. doi: 10.3390/v11070602 (PMC6669553; doi:10.3390/v11070602)
Supplement: Supplementary file 1 [file viruses-11-00602-s001.zip › Tabela S1.docx]

| **Table S1**. Genomes used in this paper for reconstruction of the baculovirus phylogeny in both the Fig. 2. The isolates placed into the genera *Alphabaculovirus* (dark blue), *Betabaculovirus* (pink), *Gammabaculovirus* (orange), and *Deltabaculovirus* (light blue) are presented together with the acronym used in the main text, the host family where the virus was isolated from, the G+C content, the Genbank accession number, the genome size, and the global identity in relation to DijuNPV (using a pairwise alignment of the concatenated nucleotide sequence of the 38 baculovirus core genes). | | | | | | | |
| --- | --- | --- | --- | --- | --- | --- | --- |
| **Baculovirus** | | **Acronym** | **Host family** | **G + C content (%)** | **Accession number** | **Genome Size (bp)** | **Id (%)** |
| 1 | Adoxophyes honmai nucleopolyhedrovirus | AdhoNPV | Tortricidae | 35.6 | AP006270 | 113220 | 46.5 |
| 2 | Adoxophyes orana nucleopolyhedrovirus | AdorNPV | Tortricidae | 35 | EU591746 | 111724 | 46.4 |
| 3 | Agrotis ipsilon multiple nucleopolyhedrovirus strain Illinois | AgipMNPV | Noctuidae | 48.6 | EU839994 | 155122 | 49.2 |
| 4 | Agrotis segetum nucleopolyhedrovirus | AgseNPV | Noctuidae | 45.7 | DQ123841 | 147544 | 48.9 |
| 5 | Antheraea pernyi nucleopolyhedrovirus isolate L2 | AnpeNPV-L2 | Saturniidae | 53.5 | EF207986 | 126246 | 72.3 |
| 6 | Anticarsia gemmatalis multiple nucleopolyhedrovirus | AgMNPV | Noctuidae | 44.5 | DQ813662 | 132239 | 72.4 |
| 7 | Apocheima cinerarium nucleopolyhedrovirus | ApciNPV | Geometridae | 33.4 | FJ914221 | 123876 | 42 |
| 8 | Autographa californica multiple nucleopolyhedrovirus clone C6 | AcMNPV-C6 | Noctuidae | 40.7 | L22858 | 133894 | 61.7 |
| 9 | Bombyx mandarina nucleopolyhedrovirus S2 | BomaNPV-S2 | Bombycidae | 40.4 | JQ071499 | 129646 | 61.3 |
| 10 | Bombyx mori nucleopolyhedrovirus strain T3 | BmNPV-T3 | Bombycidae | 40.4 | L33180 | 128413 | 61.3 |
| 11 | Buzura suppressaria nucleopolyhedrovirus | BusuNPV | Geometridae | 36.8 | KF611977 | 120420 | 47.5 |
| 12 | Catopsilia pomona nucleopolyhedrovirus | CapoNPV | Pieridae | 39.7 | KU565883 | 128058 | 60.6 |
| 13 | Choristoneura fumiferana defective multiple nucleopolyhedrovirus | CfDEFMNPV | Tortricidae | 45.8 | AY327402 | 131160 | 72.5 |
| 14 | Choristoneura fumiferana multiple nucleopolyhedrovirus | CfMNPV | Tortricidae | 50.1 | AF512031 | 129593 | 75.9 |
| 15 | Choristoneura murinana nucleopolyhedrovirus | ChmuNPV | Tortricidae | 50 | KF894742 | 124688 | 76.6 |
| 16 | Choristoneura occidentalis nucleopolyhedrovirus | ChocNPV | Tortricidae | 50.1 | KC961303 | 128446 | 75.9 |
| 17 | Choristoneura rosaceana nucleopolyhedrovirus | ChroNPV | Tortricidae | 48.6 | KC961304 | 129052 | 76 |
| 18 | Chrysodeixis chalcites nucleopolyhedrovirus | ChchNPV | Noctuidae | 39 | AY864330 | 149622 | 46.4 |
| 19 | Chrysodeixis includens single nucleopolyhedrovirus isolate IF | ChinSNPV-IF | Noctuidae | 39.2 | KU669293 | 139181 | 46.1 |
| 20 | Clanis bilineata nucleopolyhedrovirus | ClbiNPV | Sphingidae | 37.7 | DQ504428 | 135454 | 46.7 |
| 21 | Condylorrhiza vestigialis multiple nucleopolyhedrovirus | CoveMNPV | Crambidae | 42.9 | KJ631623 | 125767 | 72 |
| 22 | Cryptophlebia peltastica ucleopolyhedrovirus | CrpeNPV | Tortricidae | 37.2 | MH394321 | 115728 | 47 |
| 23 | Cyclophragma undans nucleopolyhedrovirus | CyunNPV | Lasiocampidae | 45.1 | KT957089 | 140418 | 59.3 |
| 24 | Dasychra pudibunda nucleopolyhedrovirus | DapuNPV | Lymantriidae | 54.4 | KP747440 | 136761 | 78 |
| 25 | Dendrolimus kikuchii nucleopolyhedrovirus | DekiNPV | Lasiocampidae | 48 | JX193905 | 141454 | 59.3 |
| 26 | **Dione juno nucleopolyhedrovirus** | **DijuNPV** | **‎Nymphalidae** | **50.9** | **-** | **122075** | **100** |
| 27 | Ecotropis obliqua nucleopolyhedrovirus strain A1 | EcobNPV-A1 | Geometridae | 37.6 | DQ837165 | 131204 | 47.7 |
| 28 | Epiphyas postvittana nucleopolyhedrovirus | EppoNPV | Tortricidae | 40.7 | AY043265 | 118584 | 68.9 |
| 29 | Euproctis pseudoconspersa nucleopolyhedrovirus | EupsNPV | Lymantriidae | 40.3 | FJ227128 | 141291 | 47.3 |
| 30 | Helicoverpa armigera multiple nucleopolyhedrovirus | HaMNPV | Noctuidae | 40.1 | EU730893 | 154196 | 47.5 |
| 31 | Helicoverpa armigera nucleopolyhedrovirus G4 | HaNPV-G4 | Noctuidae | 39 | AF271059 | 130759 | 46.5 |
| 32 | Helicoverpa zea single nucleopolyhedrovirus USA | HzSNPV-USA | Noctuidae | 39.1 | AF334030 | 130869 | 46.8 |
| 33 | Hemileuca sp. nucleopolyhedrovirus | HespNPV | Saturniidae | 38.1 | KF158713 | 140633 | 45.8 |
| 34 | Hyphantria cunea nucleopolyhedrovirus | HycuNPV | Arctiidae | 45.5 | AP009046 | 132959 | 75.1 |
| 35 | Hyposidra talaca nucleopolyhedrovirus | HytaNPV | Geometridae | 39.6 | MH261376 | 139089 | 48.5 |
| 36 | Lambdina fiscellaria nucleopolyhedrovirus | LafiNPV | Geometridae | 43.7 | KP752043 | 157977 | 46.1 |
| 37 | Leucania separata nuclear polyhedrovirus strain AH1 | LeseNPV-AH1 | Noctuidae | 48.6 | AY394490 | 168041 | 47.3 |
| 38 | Lonomia obliqua multiple nucleopolyhedrovirus | LoobMNPV | Saturniidae | 35.7 | KP763670 | 120022 | 57.3 |
| 39 | Lymantria dispar multiple nucleopolyhedrovirus | LdMNPV | Lymantriidae | 57.5 | AF081810 | 161046 | 50.2 |
| 40 | Lymantria xylina multiple nucleopolyhedrovirus | LyxyMNPV | Lymantriidae | 53.5 | GQ202541 | 156344 | 50 |
| 41 | Malacosoma neustria nucleopolyhedrovirus isolate T2 | ManeNPV-T2 | Lasiocampidae | 38.2 | KY968317 | 130202 | 47.8 |
| 42 | Mamestra brassicae multiple nucleopolyhedrovirus strain K1 | MbMNPV-K1 | Noctuidae | 40.1 | JQ798165 | 152710 | 47.5 |
| 43 | Mamestra configurata nucleopolyhedrovirus B | MacoNPV-B | Noctuidae | 40 | AY126275 | 158482 | 47.5 |
| 44 | Mamestra configurata nucleopolyhedrovirus-A strain 90/2 | MacoNPV-A 90/2 | Noctuidae | 41.7 | U59461 | 155060 | 47.7 |
| 45 | Maruca vitrata multiple nucleopolyhedrovirus | MaviMNPV | Crambidae | 38.6 | EF125867 | 111953 | 60.7 |
| 46 | Mythimna unipuncta nucleopolyhedrovirus strain #7 | MyunNPV#7 | Noctuidae | 48.6 | MF375894 | 148482 | 48.7 |
| 47 | Mythimna unipuncta nucleopolyhedrovirus strain KY310 | MyunNPV-KY310 | Noctuidae | 43.9 | MH124167 | 156647 | 46.4 |
| 48 | Operophtera brumata nucleopolyhedrovirus | OpbuNPV | Geometridae | 38.9 | MF614691 | 119054 | 44.4 |
| 49 | Orgyia leucostigma nucleopolyhedrovirus isolate CFS-77 | OrleNPV | Lymantriidae | 39.9 | EU309041 | 156179 | 48 |
| 50 | Orgyia pseudotsugata multiple nucleopolyhedrovirus | OpMNPV | Lymantriidae | 55.1 | U75930 | 131995 | 77.9 |
| 51 | Oxyplax ochracea nucleopolyhedrovirus | OxocNPV | Limacodidae | 31.2 | MF143631 | 113971 | 54.8 |
| 52 | Peridroma sp. nucleopolyhedrovirus | PespNPV | Noctuidae | 53.2 | KM009991 | 151109 | 50.5 |
| 53 | Perigonia lusca single nucleopolyhedrovirus | PeluSNPV | Sphingidae | 39.6 | KM596836 | 132831 | 47.8 |
| 54 | Philosamia cynthia ricini nucleopolyhedrovirus | PhcyNPV | Saturniidae | 53.7 | JX404026 | 125376 | 72 |
| 55 | Plutella xylostella multiple nucleopolyhedrovirus isolate CL3 | PlxyMNPV | Plutellidae | 40.7 | DQ457003 | 134417 | 61.9 |
| 56 | Rachiplusia ou multiple nucleopolyhedrovirus | RoMNPV | Noctuidae | 39.1 | AY145471 | 131526 | 61.4 |
| 57 | Spilosoma obliqua nucleopolyhedrosis virus isolate IIPR | SpobNPV-IIPR | Erebidae | 45.5 | KY550224 | 136141 | 75 |
| 58 | Spodoptera exempta nucleopolyhedrovirus strain 244.1 | SpexNPV-244.1 | Noctuidae | 41.2 | MH717816 | 129528 | 47.7 |
| 59 | Spodoptera exigua multiple nucleopolyhedrovirus | SeMNPV-US1 | Noctuidae | 43.8 | AF169823 | 135611 | 48.2 |
| 60 | Spodoptera exigua multiple nucleopolyhedrovirus isolate QD | SeMNPV-QD | Noctuidae | 37.4 | MH370144 | 128525 | 47 |
| 61 | Spodoptera frugiperda multiple nucleopolyhedrovirus isolate 19 | SfMNPV-I19 | Noctuidae | 40.3 | EU258200 | 132565 | 47.8 |
| 62 | Spodoptera litoralis nucleopolyhedrovirus isolate AN1956 | SpliNPV-1956 | Noctuidae | 44.7 | JX454574 | 137998 | 46 |
| 63 | Spodoptera litura multiple nucleopolyhedrovirus G2 | SlMNPV-G2 | Noctuidae | 42.8 | AF325155 | 139342 | 45.9 |
| 64 | Spodoptera litura nucleopolyhedrovirus II | SliNPV-II | Noctuidae | 45 | EU780426 | 148634 | 48.5 |
| 65 | Sucra jujuba nucleopolyhedrovirus | SujuNPV | Geometridae | 38.7 | KJ676450 | 135952 | 47 |
| 66 | Thysanoplusia orichalcea nucleopolyhedrovirus | ThorNPV | Noctuidae | 39.5 | JX467702 | 132978 | 61.5 |
| 67 | Trichoplusia ni single nucleopolyhedrovirus | TnSNPV | Noctuidae | 39 | DQ017380 | 134394 | 46.4 |
| 68 | Urbanus proteus nucleopolyhedrovirus | UrprNPV | Hesperiidae | 34.7 | KR011717 | 105555 | 45.9 |
| 69 | Adoxophyes orana granulovirus | AdorGV | Tortricidae | 34.5 | AF547984 | 99657 | 36.7 |
| 70 | Agrotis segetum granulovirus-L1 | AgseGV-L1 | Noctuidae | 37.3 | KC994902 | 131442 | 36.5 |
| 71 | Artogeia rapae granulovirus isolate Wuhan | ArraGV-Wuhan | Pieridae | 33.2 | GQ884143 | 108592 | 37.2 |
| 72 | Choristoneura fumiferana granulovirus | ChfuGV | Tortricidae | 50.1 | DQ333351 | 104710 | 36.5 |
| 73 | Clostera anastomosis granulovirus | CaLGV | Notodontidae | 46.7 | KC179784 | 101818 | 37.7 |
| 74 | Clostera anastomosis granulovirus isolate ClanGV-B | ClanGV-B | Notodontidae | 37.8 | KR091910 | 107409 | 37.4 |
| 75 | Clostera anachoreta granulovirus | ClanGV | Notodontidae | 44.4 | HQ116624 | 101487 | 37.3 |
| 76 | Cnaphalocrocis medinalis granulovirus | CnmeGV | Crambidae | 35.2 | KP658210 | 112060 | 36 |
| 77 | Cryptophlebia leucotreta granulovirus isolate CV3 | CrleGV | Tortricidae | 32.4 | AY229987 | 110907 | 36.4 |
| 78 | Cydia pomonella granulovirus | CpGV | Tortricidae | 45.3 | U53466 | 123500 | 38.2 |
| 79 | Diatraea saccharalis granulovirus | DisaGV | Crambidae | 34.9 | KP296186 | 98392 | 36.9 |
| 80 | Epinotia aporema granulovirus | EpapGV | Tortricidae | 41.5 | JN408834 | 119082 | 38.5 |
| 81 | Erinnyis ello granulovirus | ErelGV | Sphingidae | 38.7 | KJ406702 | 102759 | 37.6 |
| 82 | Helicoverpa armigera granulovirus | HaGV | Noctuidae | 40.8 | EU255577 | 169794 | 37.7 |
| 83 | Mocis latipes granulovírus | MolaGV | Noctuidae | 38.3 | KR011718 | 134272 | 37.5 |
| 84 | Mythimna unipuncta granulovírus | MyunGV#8 | Noctuidae | 49.9 | KX855660 | 144673 | 39.3 |
| 85 | Phthorimaea operculella granulovirus | PhopGV | Gelechiidae | 35.7 | AF499596 | 119217 | 37 |
| 86 | Pieris rapae granulovirus isolate E3 | PiraGV-E3 | Pieridae | 33.2 | GU111736 | 108476 | 37.3 |
| 87 | Plodia interpunctella granulovirus | PiGV | Pyralidae | 44.2 | KX151395 | 112536 | 38.2 |
| 88 | Plutella xylostella granulovirus | PlxyGV | Plutellidae | 40.7 | AF270937 | 100999 | 37.9 |
| 89 | Pseudaletia unipuncta granulovirus strain Hawaiin | PsunGV-Hawaiin | Noctuidae | 39.8 | EU678671 | 176677 | 37.4 |
| 90 | Spodoptera frugiperda granulovirus isolate VG008 | SpfrGV-VG008 | Noctuidae | 46.2 | KM371112 | 140913 | 39 |
| 91 | Spodoptera litura granulovirus isolate K1 | SpliGV | Noctuidae | 38.8 | DQ288858 | 124121 | 36.3 |
| 92 | Trichoplusia ni granulovirus | TnGV | Noctuidae | 39.8 | KU752557 | 175360 | 37.4 |
| 93 | Xestia c-nigrum granulovirus | XcGV | Noctuidae | 40.7 | AF162221 | 178733 | 37.6 |
| 94 | Neodiprion abietis nucleopolyhedrovirus | NeabNPV | Diprionidae | 33.4 | DQ317692 | 84264 | 32.8 |
| 95 | Neodiprion lecontei nucleopolyhedrovirus | NeleNPV | Diprionidae | 33.4 | AY349019 | 81755 | 32.9 |
| 96 | Neodiprion sertifer nucleopolyhedrovirus | NeseNPV | Diprionidae | 33.8 | AY430810 | 86462 | 33 |
| 97 | Culex nigripalpus nucleopolyhedrovirus | CuniNPV | Culicidae | 50.9 | AF403738 | 108252 | 30.1 |
