# Supplementary material for: A Nymphalid-Infecting Group I Alphabaculovirus Isolated from the Major Passion Fruit Caterpillar Pest Dione juno juno (Lepidoptera: Nymphalidae)
Source: Viruses. 2019 Jul 3;11(7):602. doi: 10.3390/v11070602 (PMC6669553; doi:10.3390/v11070602)
Supplement: Supplementary file 1 [file viruses-11-00602-s001.zip › Table S3.docx]

| **Table S3**. SNPs found in the DijuMNPV genome | | | | |
| --- | --- | --- | --- | --- |
| **ORF** | **Variant** | **Frequency** | **Position** | **Protein Effect** |
| polh | A | 41.4 | 171 | None |
| pk-1 | T | 41.0 | 2645 | R -> W |
| pe38 | T | 40.5 | 3345 | None |
|  | C | 31.4 | 3575 | S -> A |
| DijuMNPV-ORF-6 | T | 37.8 | 4023 | None |
|  | A | 38.4 | 4093 | D -> N |
|  | T | 40.2 | 4174 | L -> F |
| ie-2 | A | 40.6 | 5060 | None |
| DijuMNPV-ORF-9 | T | 37.8 | 8090 | R -> C |
| DijuMNPV-ORF-10 | G | 33.3 | 8462 | R -> G |
| odv-e56 (pif-5) | A | 38.0 | 9281 | none |
|  | A | 38.0 | 9282 | G -> S |
|  | T | 37.7 | 9460 | S -> F |
|  | T | 37.8 | 9461 | S -> F |
| ie-1 | T | 39.9 | 10029 | D -> N |
|  | T | 29.3 | 10168 | None |
|  | T | 39.9 | 11138 | R -> Q |
| DijuMNPV-ORF-14 | A | 41.9 | 12395 | None |
| ie-0 | C | 37.9 | 15546 | None |
| me53 | T | 41.4 | 19411 | None |
| DijuMNPV-ORF-25 | C | 32.5 | 19745 | L -> P |
| p74 (pif-0) | G | 40.8 | 21351 | I -> M |
|  | T | 39.9 | 21945 | None |
|  | A | 38.6 | 22002 | None |
|  | A | 43.5 | 22108 | E -> K |
| p10 | C | 31.2 | 22501 | P -> A |
| p26 | T | 31.8 | 23081 | D -> N |
| alk-exo | T | 31.8 | 24642 | None |
| ac132-like | G | 37.7 | 25736 | none |
|  | G | 37.7 | 25737 | Y -> S |
|  | G | 40.0 | 26081 | None |
| pep | C | 43.3 | 26425 | None |
|  | A | 39.7 | 26671 | None |
|  | A | 39.6 | 26698 | None |
| gp64 | A | 42.4 | 28748 | None |
|  | T | 41.2 | 28777 | None |
| vchi | T | 39.8 | 31401 | None |
|  | C | 42.0 | 31578 | None |
|  | C | 39.0 | 31653 | None |
|  | A | 30.2 | 32454 | None |
| DijuMNPV-ORF-39 | A | 29.9 | 32965 | None |
|  | G | 41.0 | 33024 | F -> L |
|  | A | 30.9 | 33029 | A -> V |
| lef-7 | G | 41.0 | 33024 | none |
|  | A | 30.9 | 33029 | C -> Y |
| ac120-like | C | 42.5 | 34976 | I -> V |
|  | C | 42.3 | 35062 | Q -> R |
| pif-1 | T | 31.6 | 35412 | R -> Q |
|  | A | 37.4 | 35504 | None |
|  | A | 37.0 | 35513 | None |
|  | C | 42.2 | 35618 | None |
| ac114-like | A | 35.6 | 38782 | S -> N |
|  | C | 36.2 | 38801 | None |
|  | G | 39.5 | 39089 | None |
| ac110-like | G | 39.6 | 39900 | None |
|  | T | 29.4 | 40012 | R -> K |
|  | A | 42.4 | 40016 | P -> S |
| DijuMNPV-ORF-52 | T | 29.4 | 40012 | D -> N |
|  | A | 42.4 | 40016 | none |
|  | G | 40.8 | 40070 | None |
| odv-ec43 | A | 41.2 | 40478 | None |
|  | A | 38.7 | 41072 | None |
|  | G | 39.2 | 41361 | N -> D |
| ac108-like | T | 32.4 | 41710 | T -> I |
|  | T | 38.5 | 41731 | S -> F |
| ac106/107-like | G | 37.4 | 42403 | None |
|  | T | 38.0 | 42418 | None |
| vp80 | A | 32.6 | 43518 | R -> L |
| p48 | A | 40.2 | 45994 | A -> T |
| p12 | A | 41.4 | 46414 | None |
| p40 | A | 39.6 | 47137 | None |
| 38k | C | 40.7 | 49519 | None |
| dna-helicase | A | 42.6 | 50985 | A -> T |
|  | T | 38.8 | 51374 | None |
|  | T | 31.6 | 52802 | None |
|  | A | 31.7 | 52934 | None |
| p33 | A | 40.6 | 55827 | None |
| ac91-like | C | 43.5 | 56546 | None |
| lef-4 | A | 39.9 | 56650 | None |
|  | T | 38.8 | 56703 | V -> M |
|  | T | 37.2 | 56866 | None |
|  | G | 38.0 | 56884 | None |
|  | A | 39.0 | 56995 | None |
|  | T | 39.5 | 57253 | None |
|  | T | 40.4 | 57633 | E -> K |
| vp39 | C | 42.4 | 58340 | None |
|  | T | 38.9 | 58444 | None |
|  | T | 39.9 | 58880 | None |
|  | A | 40.2 | 58961 | D -> N |
| vp91 | C | 42.0 | 61107 | K -> E |
|  | A | 40.9 | 61859 | T -> I |
|  | T | 40.0 | 61966 | None |
|  | G | 39.6 | 62217 | S -> P |
| ac82-like | C | 38.2 | 62955 | V -> A |
|  | A | 38.4 | 62990 | S -> T |
| gp41 | T | 38.3 | 64278 | None |
|  | T | 38.4 | 64279 | None |
| ac79-like | G | 36.8 | 65023 | N -> S |
|  | A | 43.7 | 65205 | E -> K |
| ac78-like | T | 31.8 | 65612 | P -> S |
| ac76-like | C | 31.0 | 66919 | V -> A |
| ac75-like | A | 32.8 | 67162 | A -> T |
| ac74-like | G | 21.0 | 67510 | N -> D |
|  | T | 19.9 | 67530 | None |
|  | T | 21.5 | 67565 | A -> V |
|  | A | 40.0 | 67670 | R -> Q |
| iap-2 | A | 40.5 | 69237 | None |
| lef-3 | T | 37.7 | 71249 | E -> D |
|  | G | 39.1 | 71650 | N -> S |
| desmoplakin | T | 38.0 | 71740 | D -> N |
|  | G | 38.8 | 72236 | None |
|  | T | 38.8 | 73456 | A -> T |
|  | T | 39.7 | 73638 | S -> N |
| dna-pol | C | 39.2 | 74497 | None |
|  | C | 43.0 | 74529 | L -> S |
|  | C | 43.4 | 74618 | None |
|  | A | 32.3 | 74773 | None |
|  | T | 39.4 | 75010 | None |
|  | C | 40.6 | 75367 | None |
|  | T | 40.4 | 76903 | None |
|  | G | 32.8 | 77007 | Q -> R |
|  | C | 31.0 | 77065 | None |
|  | G | 29.8 | 77086 | D -> E |
| gp37 | A | 37.5 | 77276 | None |
|  | A | 32.5 | 77337 | R -> S |
|  | A | 40.1 | 77340 | D -> N |
|  | C | 43.1 | 77680 | L -> S |
|  | G | 41.6 | 77696 | None |
|  | T | 37.9 | 77774 | None |
|  | G | 34.0 | 77822 | None |
|  | C | 34.1 | 77872 | F -> S |
|  | G | 37.4 | 77905 | Q -> R |
|  | A | 43.0 | 78194 | M -> I |
| bro-a | A | 41.9 | 78251 | H -> N |
| bro-b | T | 40.4 | 78803 | E -> D |
|  | A | 43.8 | 79122 | G -> S |
| lef-9 | G | 45.5 | 81296 | V -> A |
| fp-25k | T | 40.8 | 81721 | None |
|  | A | 41.7 | 81892 | None |
|  | C | 25.6 | 82060 | None |
| chaB-2 | A | 31.0 | 82904 | S -> N |
|  | A | 33.9 | 82928 | R -> K |
|  | G | 33.8 | 82949 | P -> R |
| ac57-like | G | 43.0 | 83139 | None |
|  | T | 41.7 | 83512 | None |
| ac56-like | A | 41.5 | 83800 | None |
|  | A | 43.6 | 83953 | None |
| vp1054 | T | 40.7 | 84932 | None |
| lef-8 | A | 35.9 | 88469 | A -> D |
| gta | A | 40.9 | 91390 | None |
|  | C | 41.4 | 91423 | None |
| pkip | C | 47.9 | 94838 | M -> V |
|  | T | 44.6 | 94839 | None |
| bdp | A | 40.2 | 95528 | P -> S |
|  | C | 42.5 | 95682 | None |
|  | A | 40.5 | 96051 | None |
| iap-1 | A | 43.3 | 96581 | None |
| lef-6 | A | 36.7 | 97557 | None |
|  | A | 36.8 | 97558 | D -> N |
| ac29-like | A | 29.9 | 97896 | R -> Q |
|  | T | 32.8 | 98189 | R -> C |
| ac30-like | T | 32.8 | 98189 | D -> N |
| DijuMNPV-ORF-121 | A | 43.5 | 100062 | S -> F |
| 39k | A | 40.5 | 104645 | None |
| lef-11 | A | 34.8 | 105118 | None |
| fp | C | 38.0 | 106156 | I -> M |
|  | T | 38.5 | 106222 | None |
| arif-1 | A | 35.4 | 110200 | S -> N |
| ac19-like | G | 38.1 | 110633 | C -> R |
| ac18-like | A | 38.8 | 111548 | None |
| ac17-like | A | 39.0 | 112456 | L -> F |
| egt | A | 36.3 | 114661 | None |
|  | A | 36.8 | 114835 | None |
| DijuMNPV-ORF-143 | C | 39.1 | 117990 | H -> R |
| ac4-like | G | 44.5 | 119404 | L -> S |
| ac5-like | A | 42.6 | 119959 | A -> V |
|  | C | 45.9 | 120022 | P -> R |
| lef-2 | T | 34.0 | 120396 | V -> F |
|  | A | 33.6 | 120683 | None |
| ac45-like | T | 38.6 | 121222 | M -> I |
