# Supplementary figures and images for: A Nymphalid-Infecting Group I Alphabaculovirus Isolated from the Major Passion Fruit Caterpillar Pest Dione juno juno (Lepidoptera: Nymphalidae)
Source: Viruses. 2019 Jul 3;11(7):602. doi: 10.3390/v11070602 (PMC6669553; doi:10.3390/v11070602)

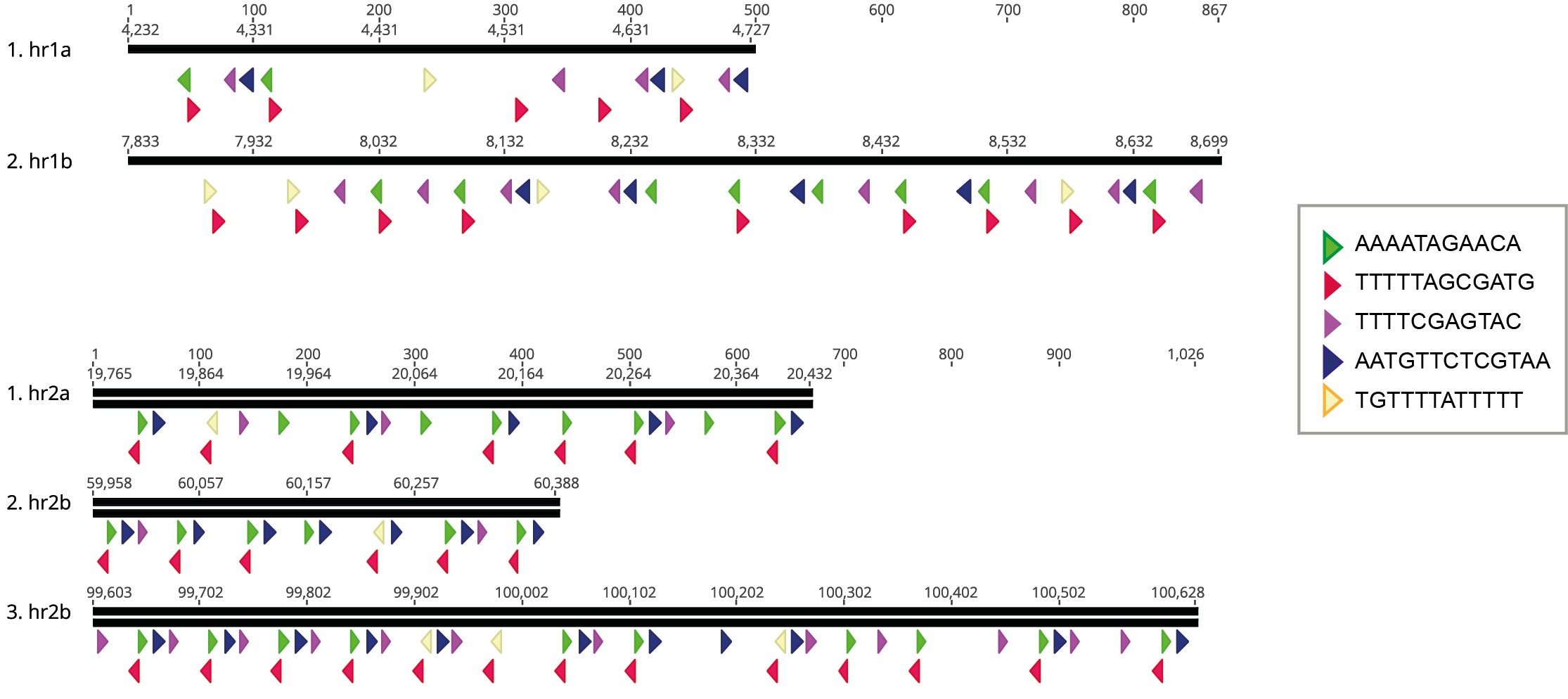

Supplement: Supplementary file 1 [file viruses-11-00602-s001.zip › Figure S1.tif]
